# Use of a novel device for intraoperative wire management during fenestrated endovascular type 4 thoracoabdominal aortic aneurysm repair

**DOI:** 10.1016/j.radcr.2024.03.084

**Published:** 2024-04-26

**Authors:** Richard L. Li, Jake Shapiro, Adam Reichard, Mark Broering, Matthew Recht, Patrick Muck

**Affiliations:** a Carle Illinois College of Medicine, University of Illinois at Urbana-Champaign; b Division of Vascular Surgery John J Cranley Vascular Laboratory, Good Samaritan Hospital Trihealth inc. Cincinnati, 375 Dixmyth Ave, Good Samaritan Hospital, Dept Graduate Medical Education, Cincinnati, OH 45233, USA

**Keywords:** Thoracoabdominal aortic aneurysm, Device, FEVAR, Medical devices, PMEG

## Abstract

Endovascular procedures are minimally invasive approaches to treat conditions affecting blood vessels without the need for large incisions. The benefits are less blood loss and faster recovery. One condition commonly treated endovascularly is aortic aneurysmal disease often secondary to atherosclerosis or chronic hypertension. As endovascular aneurysm repair becomes increasingly complex and sophisticated, the intraoperative organization and management of wires from multiple access sites becomes paramount. Often, the physician selects visceral or great vessels for delivery of stent grafts to maintain vessel patency. Loss of wire in critical target vessels and wire contamination pose significant patient risks. WireWatch (BioTex Inc. Houston, Texas, USA) is a novel device designed for intraoperative wire management to improve surgical field organization, provide wire stabilization, and prevent dropped wires. This case describes its use in a 73-year-old female undergoing a fenestrated endovascular aneurysm repair of 5.6 cm types IV thoracoabdominal aortic aneurysm.

## Introduction

New technologies and techniques have expanded the range of aortic aneurysms amenable to endovascular repair. In the US, the number of centers conducting complex endovascular aneurysm repair (EVAR) has doubled, increasing from 39 in 2014 to 81 in 2017 [1]. However, as EVARs become increasingly complex, the techniques involved become increasingly sophisticated, necessitating the use of more wires, catheters, and devices than standard EVARs. WireWatch (BioTex Inc. Houston, Texas, USA) is a sterile, single-use device designed to hold and secure guidewires during interventional procedures. This case describes the use of the WireWatch device in a complex FEVAR procedure for type IV Thoracoabdominal Aortic Aneurysm (TAA). The patient provided informed consent to the use of their case and imaging in this report.

### Case report

**Fig. 1:**
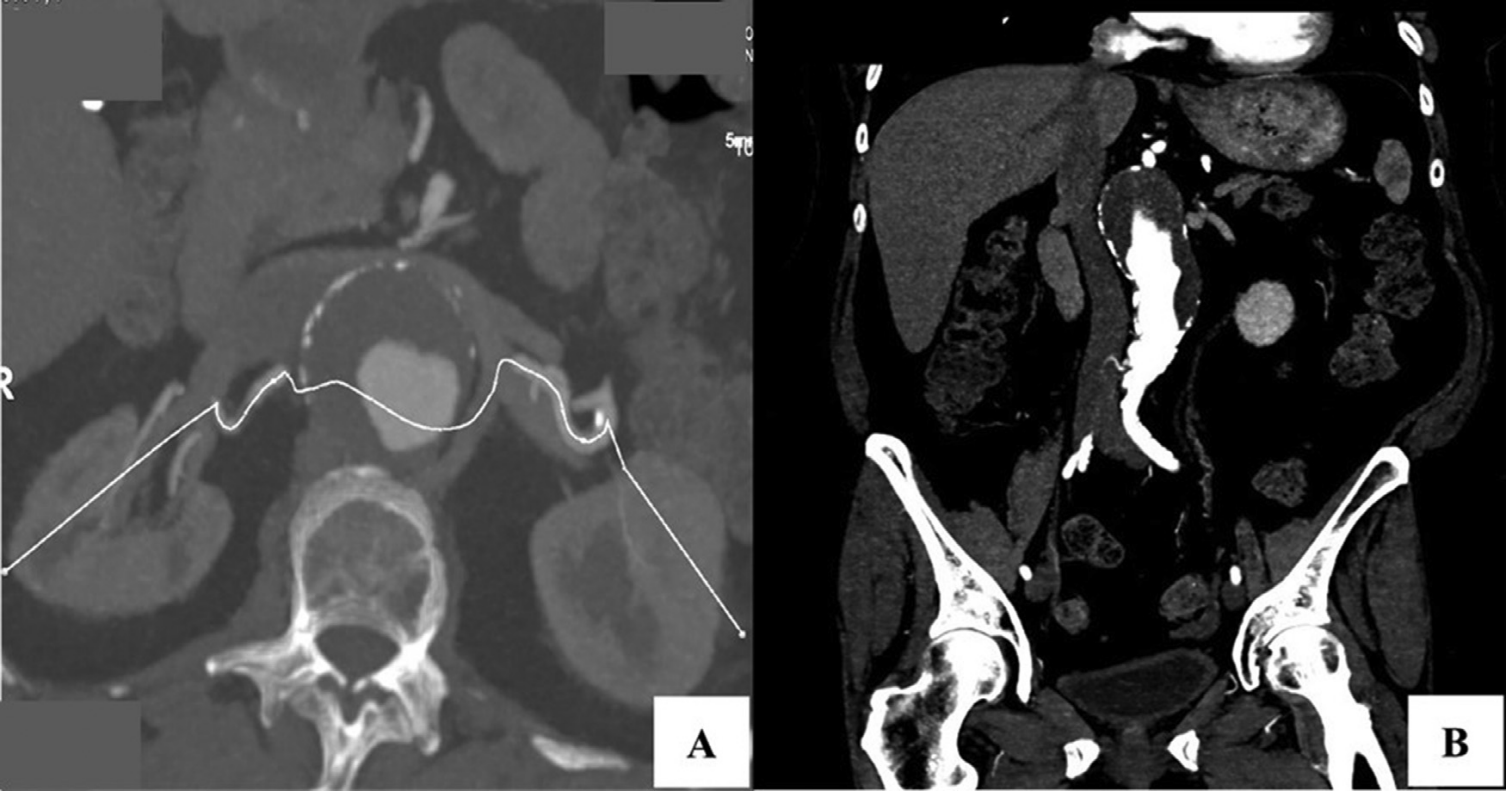
Axial (A) and coronal (B) views of preoperative CTA demonstrating a 5.6 cm Type IV TAA.

A 73-year-old female with a history of hypertension, type II diabetes, and chronic kidney disease stage 3 presented to our clinic for evaluation of a 5.6 cm type IV TAA. Prior imaging was significant for renal, SMA, and iliac artery occlusive disease. Preoperative imaging confirmed the presence of a 5.6 cm type IV TAA. Bilateral access sites were adequate, and the aneurysm tapered to a normal diameter several centimeters above the iliac bifurcation (Fig. 1). Given the anatomy and surgical risk, the decision was made to proceed with FEVAR using a physician modified aortic endograft.

### Pre-operative selective stenting

Two weeks prior to the percutaneous FEVAR, the visceral vessels were stented. These stents serve as excellent targets and, when coupled with BiPlane Fluoroscopy, ease visceral artery cannulation during FEVAR. The bilateral renal arteries were stented using 6 × 18 Cordis stents (Miami Lakes, FL, USA). The celiac artery was found to be significantly stenotic; however, the gastroduodenal artery (GDA) robustly filled the common hepatic artery. Thus, the plan was to cover the celiac. The superior mesenteric artery (SMA) was stented with an 8 × 29 Omnilink stent (Abbott Inc., Abbott Park, Illinois, USA).

### Operative technique

**Fig. 2:**
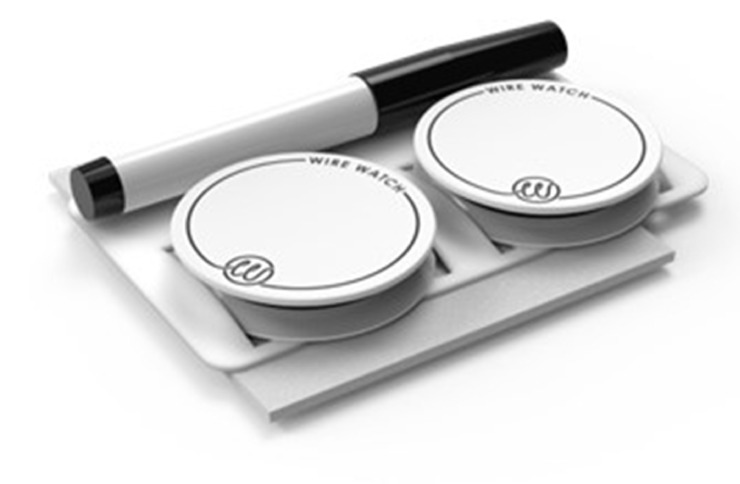
WireWatch kit including 2 devices, a marker and lint free wire wipe.

Preoperatively the patient had a spinal drain placed. The procedure was performed in the hybrid operating room using biplane fluoroscopy (Philips Inc. Amsterdam, Netherlands) under general anesthesia. To create visceral fenestrations, our preference is the 0.035 RadioFrequency (RF) PowerWire (Baylis Medical, Montreal, Quebec, Canada). Two WireWatch (BioTex Inc. Houston, Texas, USA) kits were opened on the back table. The kit includes two WireWatch devices, a sterile marker and a lint free wire cleaning wipe (Fig. 2). The WireWatch works by wedging wires between two proprietary foam disks. The bottom disk has an adhesive material that adheres to the sterile drape. This adhesive backing prevents WireWatch movement and can be placed anywhere on the field. The internal foam layer secures guidewires between 0.014 and 0.035. One WireWatch size fits all wires and prevents wire movement and wire contamination.

The bilateral common femoral arteries were accessed using a micropuncture kit under ultrasound. Two Perclose Proglides (Abbott Inc., North Chicago, IL, USA) were deployed per access site. Stiff wires were placed under fluoroscopy into the ascending aorta. An 18 and 16 French Dry seal Sheath (W.L Gore & Associates, Flagstaff, AZ, USA) were placed in the right and left access sites, respectively.

A 32/28 mm x 178 mm tapered Zenith alpha thoracic endograft (Cook Medical, Bloomington, IN, USA) was advanced through the right 18 French sheath. The graft was positioned under biplane fluoroscopy 5cm above the diseased celiac ostium and deployed. The distal end of the graft terminating in non-aneurysmal distal aorta just above the aortic bifurcation. A 16 French Aptus steerable sheath (Medtronic Inc. Minneapolis, MN, USA) was advanced through the left groin and biplane fluoroscopy was used to obtain AP and lateral views of the previously stented SMA. The RF guidewire was then advanced through the steerable sheath with the tip touching the inner lumen of the aortic endograft. Forward pressure was applied on the wire and the wire was engaged. Upon fenestration, the RF guidewire was advanced into the SMA and a 90 cm Navicross Catheter (Terumo, Shibuya City, Tokyo, Japan) was advanced into the SMA. Digital Subtraction Angiography (DSA) was performed to confirm true lumen and a 260 cm short tip Amplatz Super Stiff guidewire was placed through the Navicross catheter. A WireWatch was then placed 5 cm distal to the end of the sheath and a second one approximately 120 cm at the end of the Amplatz. The wire was secured with the novel wire securing system which is described below. The wire is removed and replaced into the WireWatch during device exchanges. A 4 × 20 mm Armada balloon (Abbott, Inc., North Chicago, IL, USA) was inflated, dilating the fenestration. The fenestration was ultimately stented with an 8 × 29 mm VBX balloon-expandable stent (W.L Gore & Associates, Flagstaff, AZ, USA), and a 10 × 20 mm balloon was used to flare the proximal stent. DSA confirmed successful stent placement and SMA perfusion.

**Fig. 3:**
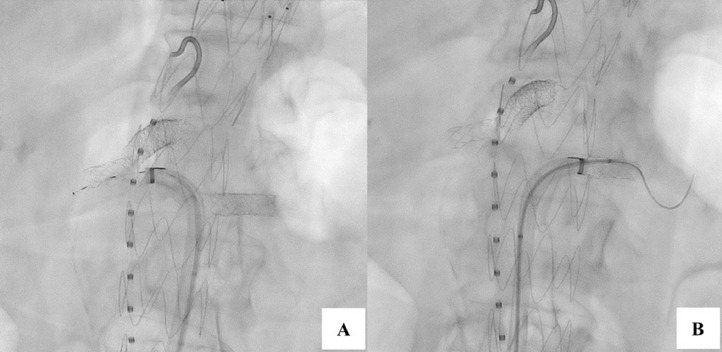
Aptus assisted RF wire fenestration of the right (A) and left (B) renal arteries.

**Fig. 4:**
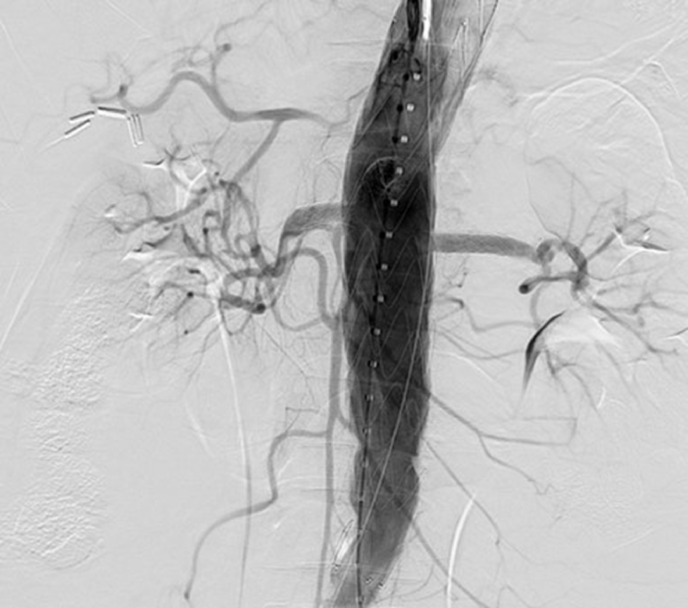
Completion angiography with no endoleaks, visceral patency with filling of the hepatic arteries from the GDA.

Attention was then turned to the bilateral renal arteries. The same technique was used with an Aptus sheath to fenestrate, cannulate, and stent the bilateral renal arteries with VBXs, 7 × 19 on right and 7 × 29 on the left (Fig. 3). WireWatches were used in the same fashion as the SMA, with two placed sequentially away from the end of the sheath securing the wire. Both stents were post-dilated and flared. DSA confirmed stent positioning with no dissections, thrombosis, or embolism. A coda balloon was then used at the proximal and distal seal ones. Completion biplane angiography confirmed no endoleaks (Fig. 4). All sheathes, wires, and devices were removed and Perclose Proglide sutures were cinched down. Patient was extubated and transferred to the PACU in stable condition.

### Post procedure

Post-operatively the patient was admitted to the surgical intensive care ward. No complications were encountered at the access site. The patient's post-operative course was uneventful, and he was discharged on post-op day 6.

## Discussion

Breaks in sterile technique and wire contamination in the cardiac catheterization lab or endovascular suite can lead to infection, significantly increasing patient morbidity and mortality [2]. Even if infection is contained and dropped wires are minimized, there are additional contaminating factors in the endovascular suite. Laird et. al published a compilation of the damaging impact that foreign bodies such as lint and other fibers can have, resulting in arteritis, granulomas and hypersensitivity reactions [3]. Several authors reported abnormal findings of cotton fibers, lint and surgical sponge particles in pulmonary arteries, coronary arteries and intracranial vessels as well [4], [5], [6], [7], [8], [9], [10], [11], [12]. Whelan et. al concluded the contaminating particles were associated with delayed wound-healing of stented vessels, prolonging the period in which subacute thrombosis could occur [13].

**Fig. 5:**
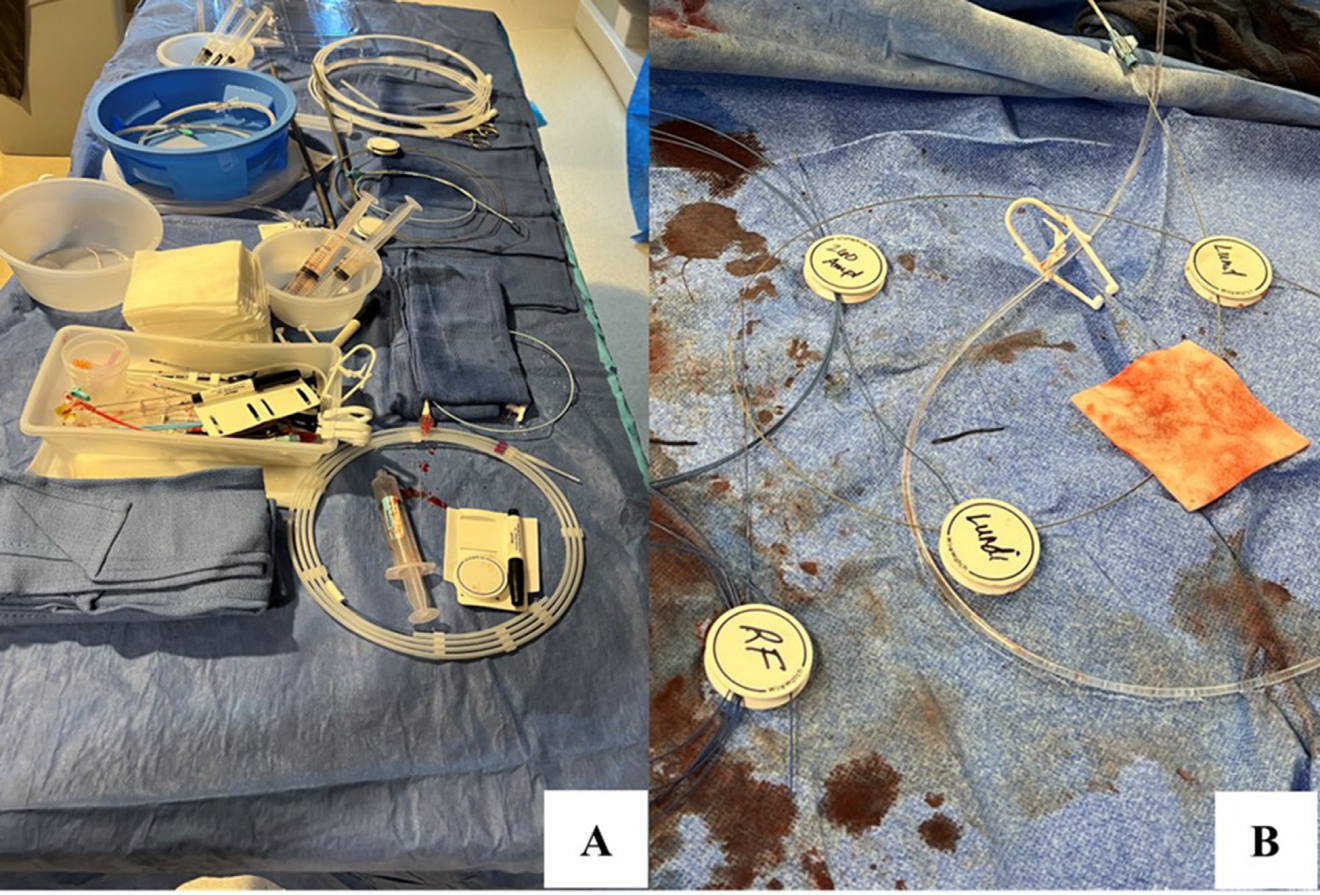
Back table (A) and endovascular table (B) wire management with several WireWatches labeled. Lint-free wipe is also shown in panel B.

This case involved the use of several different wires, devices, and catheters, each used at different points of the case. Wires were present throughout and Wire Watches were placed longitudinally down both legs to hold the wires straight when not actively in use. At one point in this procedure, there were two wires leaving each groin access site, with each set held down by the WireWatches. This was particularly helpful during DSA runs when all operators stepped away from the field. Each WireWatch comes with a marker for labelling each wire watch, allowing for organization of each wire and vessel cannulated (Fig. 5). This reduces medical towel waste and increases radiology technologist efficiency during the case.

Ultimately, the WireWatch facilitated the use of wires engaged in the patient as well as wires not in use on the back table. This case had zero dropped wires and intraoperatively no wires were mistakenly passed.

In our practice, we use WireWatches routinely to prevent dropped or damaged devices for more complex endovascular interventions. This device has proven efficacious in interventions ranging from pulmonary embolisms and deep venous thrombosis to TransCarotid Arterial Revascularization and radial interventions. Dropped or damaged wires represent an underappreciated cost concern. A study done by Wang Et. Al identified that of interventional radiologists and vascular surgeons, less than 20% were able to successfully estimate the price of devices commonly used in interventions [14]. Reviewing the costs at our institution was enlightening, with neurovascular wires costing up to $1600 per wire and our RF PowerWire costing up to $2000 per wire. Taken together, diligence and awareness of the high price of devices and efforts to improve sterility and patient outcomes should warrant further discussion and innovations of the interventional workspace.

## Conclusion

Complex endovascular aortic repair increasingly relies on the use of more devices, wires, and catheters. As these procedures and techniques become more widely adopted, the need for intraoperative organization becomes paramount. Our initial experience with the Wire Watch kit proved efficacious regarding its intended benefits: wire management, security, sterility and identification.

## Patient consent

The patient graciously provided informed consent for the use of their case and imaging in this report.
